# Inflammatory Mediators of Angiogenesis

**DOI:** 10.1155/2013/610543

**Published:** 2013-09-16

**Authors:** Grzegorz Szewczyk, Janusz Rak, Jeffrey H. Ruth

**Affiliations:** ^1^Department of General and Experimental Pathology, Medical University of Warsaw, ul. Krakowskie Przedmiescie 26/28, 00-927 Warsaw, Poland; ^2^Montreal Children's Hospital, McGill University, Canada; ^3^University of Michigan Medical School, USA

Angiogenesis gained a stable position in the field of pathogenesis. Vascular changes are a recognized hallmark of cancer growth, inflammatory disorders, and retina disorders, particularly in the context of diabetes. The inflammatory process, defined by the release of mediators from activated neutrophils, macrophages, and other myeloid cells, intersects with vascular changes, but the nature of this interrelationship frequently remains complex and puzzling, and the consequences remain difficult to predict. Clearly, a better understanding of molecular and cellular pathways that link angiogenesis and inflammation would be helpful in the development of treatment of conditions where both processes are involved. Although many of these mediators involved in angiogenesis are well known, there is still a need for further clarification of their activity. 

This special issue contains eight independent articles, of which three are focused on the pathogenesis of inadequate angiogenesis in human disease, and two are experimental papers using rodent models. There are three review articles which describe both physiological aspects of angiogenesis and its possible keys for modification.

Articles included in this special issue also reflect the notion that angiogenesis is a fragment of the biological continuum involved in tissue development, remodeling, and repair, including haemostasis, inflammation, and vascular growth. These mechanisms are often subverted in pathological states, such as chronic injury, diabetes, or cancer, but remain intimately linked. Inflammation and angiogenesis have often been studied as separate entities; however, several articles in this issue document the existence of important biological linkages between them. 

“*Fractalkine (CX3CL1) and its receptor CX3CR1 may contribute to increased angiogenesis in diabetic placenta*” by D. Szukiewicz et al. describes how an inflammatory chemokine known as fractalkine (CX3CL1) may act as direct modulator of angiogenesis by virtue of interacting with its receptors (CX3CR1) present on endothelial cells. This process is upregulated in placenta of patients with diabetes class C, leading to increased microvascular density. This is important as abnormal remodeling of placental vasculature may impact oxygen exchange and fetal health. 

“*Antiangiogenic VEGF isoform in inflammatory myopathies*” by N. Volpi et al. shows that VEGF-A165b is significantly upregulated in idiopathic inflammatory myopathies, as well as TGF-*β*. VEGF-A is also shown to be diffusely expressed on unaffected myofibres, whereas regenerating/atrophic myofibres strongly react for both VEGF-A isoforms. Most inflammatory cells and endomysial vessels express both isoforms, but VEGF-A165b levels show a positive correlation to inflammatory scores, endomysial vascularization, and TGF-*β*. These findings indicate that skeletal muscle expression of antiangiogenic VEGF-A165b and its preferential upregulation in idiopathic inflammatory myopathies suggest that modulation of VEGF-A isoforms may occur in myositides.

“*Thrombospondin and VEGF-R: is there a correlation in inflammatory bowel disease?*” by J. Wejman et al. shows significantly higher vascular density and vascular area percentage in all layers of bowel wall (not only mucosal bioptates) in patients with Crohn's disease (CD), which was evaluated by immunohistochemistry analysis. This study found differences in vascular density distribution between ulcerative colitis (CU) and CD and between CU and controls, but no statistically significant correlation between those findings and VEGFR-1 or thrombospondin-1 (TSP-1) expression. Overall, the results suggest the existence of different TSP-1 independent pathways of antiangiogenesis in inflammatory bowel disease. 

“*Inhibitory effect of herbal remedy PERVIVO and anti-inflammatory drug sulindac on L-1 sarcoma tumor growth and tumor angiogenesis in Balb/c mice*” by P. Skopiński et al. reveals the interesting observation concerning antiangiogenic properties of the herbal mixture PERVIVO. The experimental model was developed in Balb/c mice after grafting of L-1 sarcoma cells. PERVIVO shows antiangiogenic properties by decreasing de novo vessel development thereby inhibiting tumor growth. Both effects are augmented after combining PERVIVO with sulindac—in which a synergistic effect was observed.

“*The effect of anti-inflammatory and antimicrobial herbal remedy PADMA 28 on immunological angiogenesis and granulocytes activity in mice*” by D. M. Radomska-Le$\overset{´}{\text{s}}$niewska et al. was also focused on traditional medicine rationalizing the use of herbal drugs. The in vivo stimulatory effect of PADMA 28 (which is a mixture of herbal extract) for angiogenic activity can be modulated by dose and is associated with deterioration of granulocytes after high-dose treatment.

“*Cytokines and angiogenesis in the corpus luteum*” by A. M. Galvão describes angiogenesis in the corpus luteum as an extremely rapid sequence of events that determines the dramatic changes on vascular and nonvascular structures. The main purpose of this review is to highlight the interaction between immune, endothelial, and luteal steroidogenic cells, regarding vascular dynamics/changes during the establishment and regression of the equine corpus luteum.

“*Matrix metalloproteinases: inflammatory regulators of cell behaviour in vascular formation and remodelling*” by Q. Chen et al. gives the systematic review of the role of matrix metalloproteinases (MMP) in the process of angiogenesis. The main purpose of this review is to show a complex interaction between MMP stimulators, their mechanism of activity and inflammatory cell recruitment.

“*Angiogenesis and its therapeutic opportunities*” by S. Y. Yo and S. M. Kwon offers a general review of angiogenesis with a particular focus on clinical applications.

There are general implications concerning the crosstalk between inflammatory and angiogenic circuitries. For instance, effective ways to lower pathological angiogenesis may require targeting not only “professional” regulators of endothelial cell function, but also elements of the inflammatory network, a notion that has already been linked to resistance of certain cancers to antiangiogenic agents. While articles published in this special issue were not intended to fully address these questions (the area is simply too vast), they do draw attention to specific scenarios in which the inflammatory-vascular continuum is particularly manifest or functionally significant. We hope that this integrated view will be of interest and of stimulating value to the readers.

*Grzegorz Szewczyk* *Grzegorz Szewczyk* *Janusz Rak* *Janusz Rak* *Jeffrey H. Ruth* *Jeffrey H. Ruth*

